# Structural composition and evolution of jujube centromere reveal a dominant role for LTR retrotransposon

**DOI:** 10.1093/hr/uhaf244

**Published:** 2025-09-15

**Authors:** Donghui Lin, Yunxin Lan, Zhongchen Zhang, Jingjing Guo, Jian Shen, Guoliang Wang, Shufeng Zhang, Yihan Yang, Jiao Li, Guiming Liu, Zhiguo Liu, Mengjun Liu, Meng Yang

**Affiliations:** College of Horticulture, Hebei Agricultural University, Baoding 071001, Hebei, China; College of Horticulture, Hebei Agricultural University, Baoding 071001, Hebei, China; College of Horticulture, Hebei Agricultural University, Baoding 071001, Hebei, China; College of Horticulture, Hebei Agricultural University, Baoding 071001, Hebei, China; College of Horticulture, Hebei Agricultural University, Baoding 071001, Hebei, China; Institute of Biotechnology, Beijing Academy of Agriculture and Forestry Sciences, Beijing, China; College of Horticulture, Hebei Agricultural University, Baoding 071001, Hebei, China; College of Horticulture, Hebei Agricultural University, Baoding 071001, Hebei, China; College of Horticulture, Hebei Agricultural University, Baoding 071001, Hebei, China; Institute of Biotechnology, Beijing Academy of Agriculture and Forestry Sciences, Beijing, China; College of Horticulture, Hebei Agricultural University, Baoding 071001, Hebei, China; College of Horticulture, Hebei Agricultural University, Baoding 071001, Hebei, China; College of Horticulture, Hebei Agricultural University, Baoding 071001, Hebei, China

## Abstract

Centromeres are essential for centromere-specific histone H3 (CENH3) recruitment and kinetochore assembly, ensuring accurate chromosome segregation and maintaining genome stability in plants. Although extensively studied in model species, the structural organization of centromeres in nonmodel plants, such as fruit trees, remains poorly explored. Our previous study revealed that jujube centromeres lack the typical tandem repeat (TR)-rich structure, complicating their precise identification. In this study, we updated the genome assembly of jujube (*Ziziphus jujuba* Mill. ‘Dongzao’) to a haplotype-resolved T2T version, enabling accurate mapping and comparison of centromeres between haplotypes using CENH3 ChIP-seq. These centromeres, ranging from 0.75 to 1.40 Mb, are largely conserved between haplotypes, except for a localized inversion on chromosome 10. Unlike the TR-rich centromeres found in many plant species, jujube centromeres are predominantly composed of *Gypsy*-type long-terminal repeat retrotransposons (LTR-RTs). Among these, we identified a centromere-enriched LTR family, centromeric retrotransposons of jujube (CRJ), which is particularly abundant in terminal LTRs compared to the internal transposon regions. Comparative analysis across plant species revealed that centromeric retrotransposons primarily fall into three subfamilies—*CRM*, *Tekay*, and *Athila*—highlighting strong subfamily specificity. Notably, early insertions of CRJ-derived LTR segments contributed to the formation of TR-like structures, suggesting a mechanistic link between transposable elements and the evolution of centromeric tandem repeats. This work provides the first in-depth characterization of a TE-dominated centromere architecture in a fruit tree, offering new insights into the diversity and evolution of plant centromeres.

## Introduction

The centromere is a specialized chromosomal domain that plays a critical role in the segregation of sister chromatids during eukaryotic cell division. Although centromere function is broadly conserved across eukaryotes, the underlying DNA sequences evolve rapidly, leading to substantial variation in sequence composition, even among closely related species [1]. For instance, the centromere of budding yeast (*Saccharomyces cerevisiae*) is a simple ‘point’ centromere of a conserved 125 bp sequence, while that of fission yeast (*Schizosaccharomyces pombe*) spans up to 110 kb of repetitive DNA arrays [2, 3]. In contrast, plant and animal centromeres are typically much larger and structurally more diverse [4, 5].

Among eukaryotes, plants exhibit particularly wide variation in centromere organization. In most plant species, centromeric chromatin is epigenetically defined by the centromere-specific histone H3 (CENH3), which replaces canonical histone H3 in centromeric nucleosomes and serves as a master regulator of centromere identity [6]. The DNA component of plant centromeres is mainly comprised of high-copy tandem repeat (TR) units, commonly termed higher order repeats (HOR) in some well-studied plants [7]. For example, the 178 bp CEN180 in *Arabidopsis thaliana*, the 91/92/413 bp CentGm repeats in soybean (*Glycine max*), and the 176 bp Cent-SRs in *Brassica rapa* are present in thousands of copies and co-localize with CENH3 chromatin [8–10]. However, recent studies have shown that satellite repeats do not dominate all plant centromeres. In Poaceae species such as *Triticum monococcum* [11] and *Avena sativa* [12], centromeres are enriched with long-terminal repeat retrotransposons (LTR-RTs), reflecting an alternative organizational strategy. Moreover, hybrid centromeres in *Secale cereale* integrate recent centromeric retrotransposon insertions alongside degraded minisatellite sequences, underscoring ongoing structural remodeling [13]. In Solanaceae crops—including *Solanum tuberosum* [14], *Capsicum annuum* [15], and *Nicotiana tabacum* [16]—LTR-RTs exceed TRs in abundance. Together, these findings suggest that the genomic organization of plant centromeres is highly plastic during evolution.

Recent research has also revealed that certain plant centromeres harbor a class of enriched centromeric retrotransposons (CRs) that specifically bind to the CENH3 histone, potentially contributing to centromere function by modulating chromatin conformation. Through their reverse transcription and transposition, CRs can insert periodically within satellite repeat arrays that facilitate the stable assembly of CENH3 nucleosomes, and possibly maintain centromere size and increasing the repeat content of centromeres that lack TRs [4, 6]. For instance, in *A. thaliana*, *Athila* retrotransposons have been observed to integrate into centromeric TRs, disrupting the genetic and epigenetic organization of the centromere [17]. Beyond such disruptions, hypotheses regarding the origin of centromeric TRs suggest that these sequences may have arisen from the repetitive expansion of CRs [18]. For example, the maize centromeric satellite repeats CRM1TR and CRM4TR share 97% sequence similarity with segments of their corresponding LTRs, implying that an LTR-mediated copy-and-paste mechanism directly drives the expansion of satellite repeats [19]. Similar phenomena have also been observed in *Solanaceae* species, where the TRs in the centromere of potato show sequence similarity to LTR-RTs; some even retain a truncated internal region with a gag domain, surrounding by two LTRs (LTR-gag-LTR) [20, 21]. Furthermore, research in Chinese cabbage indicates that LTR insertion events in the core centromeric region (approximately 0.5 Mya) occurred significantly later than those in the pericentromeric region (>1 Mya), suggesting that continual LTR invasions may drive centromere evolution [10]. Therefore, the repetitive sequence expansion resulting from successive CR insertions may represent one of the sources of centromeric DNA in eukaryotes.

Jujube (*Ziziphus jujuba* Mill.), a fruit tree native to China with a cultivation history spanning over 7000 years, holds substantial economic and ecological importance. Its fruits are exceptionally nutritious, being rich in vitamins C and B, sugars, cyclic nucleotides, various bio-active compounds, and essential minerals—a nutritional profile aptly summarized by the traditional adage: ‘three daily jujubes delay aging’s imprints’ [22]. In 2023, we reported the first gap-free telomere-to-telomere (T2T) genome assembly of jujube using the elite ‘Dongzao’ (DZ) cultivar, primarily cultivated for fresh consumption. Initial analyses delineated 12 presumed centromeric regions through tandem repeat searches; however, the absence of a consensus core repeat unit suggests that jujube centromeres are not exclusively composed of high copy-number TRs [23]. Deciphering the precise centromere structure of jujube genome has potential benefit in understanding the chromosome segregation and genome stability, speciation and adaptation, as well as the practical meanings in breeding and crop improvement [24].

In this study, to enable precise identification and comparison of centromeres between haplotypes, we advanced the jujube (DZ) genome assembly to a haplotype-resolved level, and accurately mapped centromere positions using chromatin immunoprecipitation sequencing (ChIP-seq) data targeting the centromere-specific histone CENH3. Building on these results, we conducted comprehensive analyses to elucidate the sequence organization of jujube centromeres, assess the centromere structure differences between the two haplotypes, investigate the evolutionary patterns of centromeric LTR-RTs, and explore the mechanisms driving the centromeric diversity. Our work not only further refines the jujube genome assembly but also represents the first in-depth comprehensive analysis of centromere architecture and evolution in fruit trees, which provide new insights into chromosome biology in perennial crops.

## Results

### Refining the DZ assembly and annotation

The gap free, telomere to telomere (T2T), haplotype resolved genome assemblies of the DZ cultivar—each comprising 12 chromosomes—were generated with Hifiasm using our previously published sequencing datasets [23], which contain 28.6 Gb (70×) PacBio HiFi circular consensus sequencing (CCS) reads, 50.7 Gb (123×) Oxford Nanopore Technologies (ONT) ultra-long reads, and 43.4 Gb (105×) high-throughput chromosome conformation capture (Hi-C) data. The assembly quality was validated using Hi-C contact maps and base-level depth distribution derived from HiFi read mapping (Fig. S1). The two haplotypes, herein designated HapA and HapB, exhibit highly similar genomic features: assembly sizes of 388 versus 383 Mb; repeat contents of 55.66% versus 55.51%; and protein-coding gene counts of 33 414 versus 32 989. Genome completeness reached 99.40% for HapA and 99.30% for HapB through BUSCO evaluation, and LTR Assembly Index (LAI)—indicative of assembly continuity—were 17.65 and 17.26, respectively (Table 1). All telomeric sequences were successfully captured, ranging from 4731 bp to 74 388 bp; mean telomere length measured 18 503 bp (HapA) and 16 214 bp (HapB) on the left arm, and 17 966 bp (HapA) and 16 356 bp (HapB) on the right arm (Table S1).

**Table 1:** Genomic features between the two haplotypes.

Contents	HapA	HapB
Assembly size (bp)	387 965 287	383 652 242
Number of contigs after duplicates purging	12	12
Contig N50 (bp)	32 672 292	32 418 546
Completeness of genome BUSCO (%)	99.40	99.30
Repeat content (% of genome)	55.66	55.51
*Gypsy* elements (% of genome)	16.78	16.77
*Copia* elements (% of genome)	8.97	8.86
LAI	17.65	17.26
Number of intact LTR	2242	2147
Size of intact LTR (% of genome)	3.82	3.73
Protein-coding genes	33 414	32 989
Annotated protein-coding genes	29 525	29 103
Completeness of protein BUSCO (%)	92.90	92.80
Paralogous collinear blocks	265	233
Paralogous collinear genes (% of total genes)	14.25	13.69
Paralogous collinear regions (% of genome)	52.10	51.04

**Figure 1:**
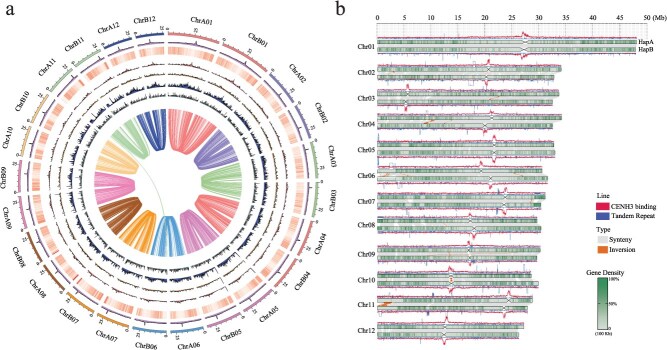
Haplotype-resolved, gap-free T2T genome assembly and centromere landscape of jujube. (a) Circos plot illustrating genomic features of the two haplotypes. Tracks from outer to inner represent: chromosomes, CENH3 ChIP-seq signal intensity, gene density heatmap, densities of *Gypsy* elements, *Copia* elements, total LTR-RTs, intact LTR-RTs, and collinearity between HapA and HapB. (b) Schematic representation of structural variations between haplotypes, along with the distribution of CENH3 Chip-seq signals (red lines, labelled as CENH3 binding) and tandem repeats (blue lines, labelled as Tandem Repeat) in 50 Kb window.

The two haplotypes exhibit highly similar chromosomal distributions of protein-coding genes and repetitive elements. Gene density progressively decreases from the telomeric regions toward the putative centromeric regions, whereas the density of repetitive sequences shows a corresponding increase (Fig. 1a). A substantial proportion of the genome is collinear between the two haplotypes, with approximately 52.10% and 51.04% of the genomic regions in HapA and HapB, respectively, showing high levels of alignment. These collinear regions encompass 14.25% genes in HapA and 13.69% genes in HapB (Table 1; Fig. 1a and b). To preliminary identify potential centromeric regions, we performed a genome-wide analysis of TR sequences. Consistent with our previous findings [23], these repeats are dispersed relatively randomly throughout the genome, and the core repeat units vary substantially in length and sequence composition (Fig. 1b), suggesting that the centromeres of jujube may adopt a noncanonical satellite repeat structure.

### Enrichment of CRs in the centromeric regions

**Figure 2:**
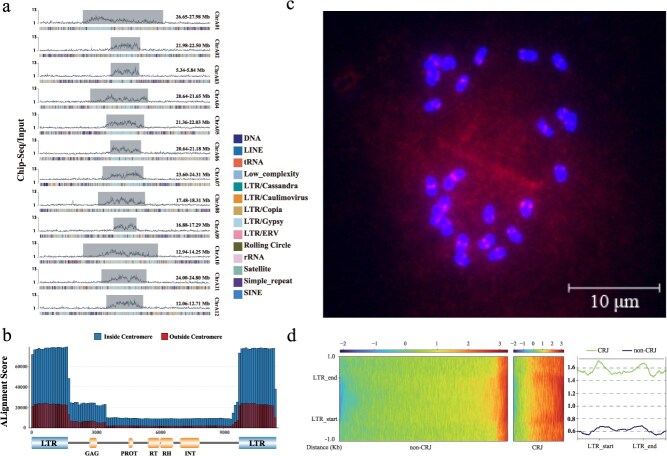
Centromere characteristics of jujube. (a) High-resolution CENH3 ChIP-seq signal profiles and repeat composition across the centromere and its flanking 2 Mb regions in the HapA genome. The line plot above represent ChIP-seq signals in 50 Kb windows, with the centromeric region shaded in dark grey. The lower stacked bar plot shows the composition and distribution of repeat types. (b) CRJ alignment scores plotted in 100 bp bins, with scores shown on the *y*-axis. The lower panel marks the structural domains of CRJ. (c) Fluorescence *in situ* hybridization (FISH) based on three CRJ-specific probes. Two distinct signals were detected on each of the two sister chromatids. (d) Comparison of ChIP enrichment between CRJ and non-CRJ (CK) LTR elements. ChIP enrichment is represented as the ratio of ChIP-seq reads to Input reads (ChIP/Input), which normalizes background noise and quantifies binding signals. The two heatmaps are based on genome-wide alignments of CRJ and non-CRJ elements and display normalized signal intensity within ±1 kb of the start and end sites of the LTRs. The line plot on the right shows the average signal profiles: green for CRJ and blue for non-CRJ elements, with the *y*-axis indicating normalized signal intensity.

To precisely delineate the centromeric boundaries, the jujube *CENH3* gene was identified through homology alignment with orthologues from other species (Fig. S2a). It is a single-copy gene encoding a protein of 152 amino acid residues. A polyclonal antibody was generated against a 20-amino acid peptide derived from this protein (Fig. S2b), and ChIP-seq was subsequently performed using this antibody. Following quality control, the ChIP-seq reads were mapped to the HapA and HapB genomes. Based on the retrieved CENH3 binding signals, centromeric regions were successfully delineated for all chromosomes in both haplotypes (Fig. 1a and b). The lengths of the identified centromeres ranged from 0.75 to 1.40 Mb across the two haplotypes (Fig. 2a; Table 2). Repeat element annotation further showed that LTR-RTs constitute a substantial portion of the centromeric regions—accounting for 84.55% in HapA and 82.02% in HapB. Notably, *Gypsy*-type LTR-RTs were particularly enriched, comprising 54.54% and 56.21% of the centromeric regions in HapA and HapB, respectively, which is markedly higher than their genome-wide proportions (HapA: 13.55%; HapB: 12.62%). This pronounced enrichment of *Gypsy* elements in the centromeric regions is consistent with patterns observed in pepper, where *Gypsy* elements account for 70.61% of LTR-RTs in the centromeres, compared to 49.67% at the whole-genome level [15] (Fig. 2a).

**Table 2:** Centromere location, composition, and FISH probe copy number in two jujube haplotypes.

Chr name	Start (Mb)	End (Mb)	Size (Kb)	Gypsy (%)	Copia (%)	TR (%)	No.^**a**^	Length^**b**^
ChrA01	26.66	28.07	1406	56.33	23.64	5.87	28	10 528
ChrA02	20.31	20.86	550	50.50	18.18	5.97	11	4136
ChrA03	5.35	5.86	517	47.30	27.30	3.22	12	4512
ChrA04	20.76	21.59	829	45.26	20.10	3.17	18	6768
ChrA05	21.30	21.99	688	66.37	17.14	2.59	16	6016
ChrA06	18.92	19.49	565	59.62	23.42	3.30	15	5640
ChrA07	23.30	24.00	693	60.26	19.01	4.73	21	7896
ChrA08	16.83	17.57	747	44.94	24.48	4.76	18	6768
ChrA09	16.65	17.08	426	51.52	17.62	2.25	16	6016
ChrA10	13.06	14.14	1080	47.88	32.28	2.23	13	4888
ChrA11	23.93	24.68	751	63.80	17.15	2.00	34	12 784
ChrA12	12.12	12.88	758	43.47	23.32	2.03	12	4512
ChrB01	26.44	27.83	1388	59.27	21.27	2.74	31	11 656
ChrB02	19.87	20.46	590	55.25	15.73	2.75	25	9400
ChrB03	5.01	5.51	499	52.64	24.12	3.03	10	3760
ChrB04	19.49	20.31	811	47.78	19.07	3.05	23	8648
ChrB05	21.27	21.95	684	66.86	14.98	3.06	17	6392
ChrB06	20.68	21.25	566	64.35	16.52	4.58	19	7144
ChrB07	23.03	23.84	804	65.21	14.33	3.88	27	10 152
ChrB08	17.55	18.27	720	55.33	18.18	2.70	25	94,00
ChrB09	16.89	17.32	426	63.33	14.57	2.43	11	4136
ChrB10	13.18	14.19	1002	54.65	23.79	2.37	19	7144
ChrB11	23.07	23.76	685	63.08	14.79	3.43	30	11 280
ChrB12	11.94	12.69	754	48.82	17.76	3.43	12	4512

a Copy number of the 376 bp sequence used for probe design (Fig. S5) on each chromosome. A mismatch threshold of 3% was allowed in the alignment.

b Total cumulative length of all copies of the 376 bp sequence on each chromosome.

Given the absence of classical satellite repeats in the centromeric regions and the dominance of *Gypsy*-type LTR-RTs, we hypothesize that centromere function in jujube may be maintained through a mechanism similar to that mediated by CRs as described in pepper [15]. To explore this possibility, we used the HapA genome as a representative and identified 13 repeat families by RepeatMasker software that occurred more than 100 times within the centromeric regions. Genome-wide alignment of these families revealed that repeat-78 and repeat-4 were predominantly enriched in centromeres. Notably, repeat-4 was entirely encompassed within the repeat-78 (12 281 bp) (Fig. S3). Based on this observation, repeat-78 was selected as a candidate sequence for identifying centromere-specific LTR-RTs. We then aligned repeat-78 to all 76 intact LTR-RTs identified in the centromeric regions across the 12 chromosomes. Among these, 48 LTR-RTs showed strong alignment with repeat-78 and were defined as CRs of jujube (CRJs). From these 48 CRJs, the element with a length of 11 454 bp—closest to that of repeat-78—was selected as the representative CRJ, which is hereafter referred to as ‘CRJ’ in this study. Subsequently, the CRJ was aligned to the whole genome, and results displayed that in addition to the 48 centromeric copies, 15 additional copies were identified, most of which were located in pericentromeric regions (Table S2). Furthermore, alignment of the 5′ and 3′ LTRs of CRJ to the genome revealed a significantly higher copy number in centromeric regions compared to the internal retrotransposon domain (Figs 2b and S4), highlighting the preferential accumulation of terminal LTR sequences within the centromeres.

Based on these findings, we selected a 376 bp sequence from the LTR region of CRJ, which represents a high-copy, centromere-specific fragment. Using this sequence, we designed three 5′-end TARMA-labeled probes (a red fluorophore) for experimental validation (Fig. S5). These probes were applied in fluorescence *in situ* hybridization (FISH) to validate the centromere location. On metaphase chromosomes, the three probes produced combined centromere-specific signals on all 24 chromosomes, with signals clearly visible on both sister chromatids. This confirmed the spatial colocalization of CRJ elements with centromeric chromatin (Figs 2c and S6). Finally, to further explore the association between CRJ elements and CENH3 binding sites, we quantified ChIP-seq enrichment by aligning CRJ and a non-CRJ element (an LTR-RT located in centromeric region but not classified as CRJs, used as control) to the genome and calculating the ratio of ChIP-seq reads to Input reads one each aligned position. The results showed that CRJ elements exhibited stronger enrichment signals compared to those of non-CRJs. Furthermore, signal intensity was higher in the LTR regions than in the internal retrotransposon domains, as indicated by stronger signals near the LTR start and end sites in the heatmaps and the pronounced peaks in the average signal line plot (green line) (Fig. 2d). These findings suggest that CRJ elements are more highly enriched in centromeric regions, and its LTRs are more closely associated with CENH3-bound chromatin than the internal domains of retrotransposons.

### Characteristics of jujube centromeres in two haplotypes

**Figure 3:**
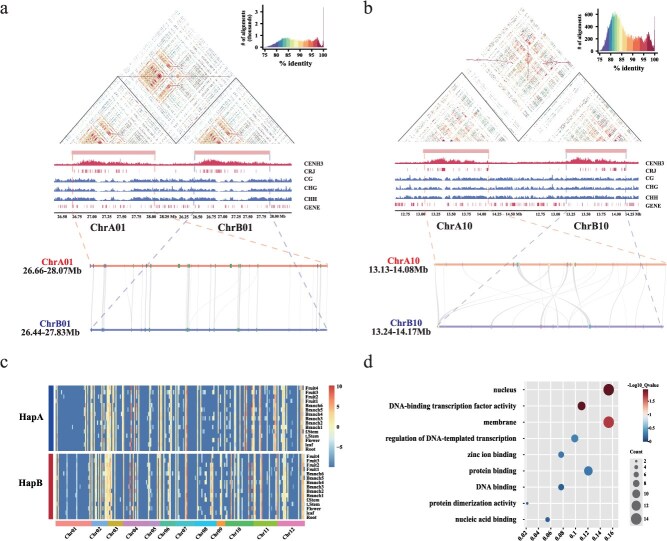
Genetic and epigenetic landscape of centromeres between two jujube haplotypes. (a) Epigenetic features of chromosome 1 comparing HapA and HapB. From top to bottom: sequence similarity matrix between HapA and HapB, intra-haplotype similarity matrices for HapA and HapB, CENH3 ChIP-seq peaks, CRJ alignment positions, DNA methylation levels in CG, CHG, and CHH contexts, gene density, and gene collinearity between the two haplotypes. (b) Epigenetic features of chromosome 10 between HapA and HapB, presented in the same order as in panel (a). (c) Expression heatmap of all genes located within centromeric regions across 15 jujube samples. Expression levels were quantified as FPKM, normalized using Z-scores, and ordered according to genomic position. (d) GO enrichment analysis of centromeric genes with total FPKM values exceeding 100 across 15 tissues in both haplotypes.

To investigate the characteristics of jujube centromeres, we extracted 2 Mb regions from each chromosome of both haplotypes, comprising the centromere and equal-length flanking pericentromeric regions. Whole-genome bisulfite sequencing (WGBS) data, which we reported previously [23], were used to analyze DNA methylation patterns across these regions. Most chromosomes exhibited high methylation levels in their centromeric regions, except for chromosome 1 in both HapA and HapB, which showed a notable reduction in methylation in the central portion of the centromere (Figs 3a and S7).

Interestingly, CRJs on chromosome 1 were predominantly located on the left side of the centromere, corresponding to regions with higher sequence identity compared to the right side. Similarly, sequence similarity between HapA and HapB within the centromere was asymmetrical, with higher identity observed in the left part than in the right (Fig. 3a). On chromosome 10, we identified a notable inversion (0.48 Mb) within the centromeric region between the two haplotypes, resulting in reverse complementary alignment of the sequences (Figs 1b, 3b, and S7). This inversion lies entirely within the centromeric region and does not displace or disrupt CENH3 positioning in either haplotype, suggesting that it does not affect centromere stability or function (Fig. S8). Examination of the remaining 10 chromosomes also revealed alignment biases within CRJ-rich regions, which consistently showed higher interhaplotype similarity than adjacent non-CRJ regions (e.g. chromosomes 4 and 6; Fig. S7). These findings suggest that CRJs exhibit unique sequence features and may play a distinct role in centromere formation and evolution.

Next, we compared gene expression level in the centromeric regions between HapA and HapB. A total of 229 and 228 protein-coding genes were identified within the centromeric regions of HapA and HapB, respectively. Transcriptome data from 15 tissues, as reported in our previous study [23], were used to profile gene expression. Overall, gene expression levels did not differ significantly between the two haplotypes (Fig. 3c). Among these genes, 158 (HapA) and 161 (HapB) were lowly expressed with FPKM values below 1. Only four genes exhibited high expression levels (FPKM >1000), annotated as glutaredoxin, fasciclin-like arabinogalactan proteins, VQ motif-containing proteins, and purple acid phosphatase 2, all of which are associated with plants’ environmental adaptation and stress/defense responses.

Chromosome-specific differences in centromeric gene expression were also observed, with centromeric genes on chromosomes two and three showing relatively higher expression levels than those on other chromosomes (Fig. 3c). Gene ontology (GO) enrichment analysis of centromeric genes uncovered several prominent functional categories. The most highly represented terms were nucleus-related processes (14 genes) and DNA-binding transcription factor activity (10 genes), indicating a central role in the regulation of gene expression and nuclear function. Nucleic acid binding (14 genes) and protein binding (12 genes) were also significantly enriched, suggesting widespread involvement in DNA–protein interactions. Although zinc ion binding (6 genes) and membrane-associated functions (8 genes) appeared less frequently, their presence points to potential modulatory roles. Interestingly, canonical centromere-specific terms were absent; instead, pathways involved in DNA repair and chromatin remodeling were prevalent. This pattern implies that centromere maintenance may rely on indirect mechanisms—such as heterochromatin formation and the mitigation of replication stress—to preserve stability (Fig. 3d).

### Evolutionary features of LTR retrotransposons in centromeric regions

Although centromeres are functionally conserved across plant species, their underlying DNA sequences vary substantially. To investigate this diversity, we analyzed centromeric regions of eight plant species—*Ananas comosus* (pineapple), *A. thaliana*, *C. annuum* (pepper), *G. max* (soybean), *Oryza sativa* (rice), *Vigna unguiculata* (cowpea), *Vitis vinifera* (grape), and *Zea mays* (maize)—in addition to jujube. The centromeric regions from these species, previously validated through ChIP-seq data, were extracted from their respective genomes for cross-species structural comparisons.

**Figure 4:**
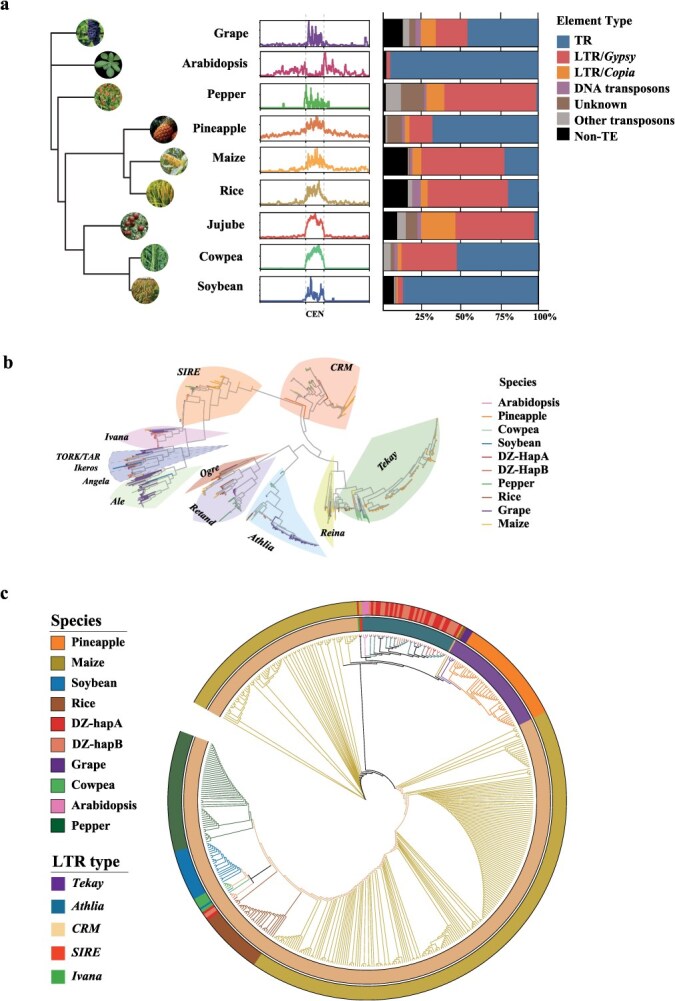
Comparative analysis of centromere structure and evolution across nine species. (a) Phylogenetic tree based on the CENH3 protein and centromere composition in nine species. From left to right: (1) Phylogenetic tree constructed using the CENH3 gene of each species; (2) Mapping of CRs from each species to its own centromere region and adjacent 2 Mb flanking regions. Each rectangle represents the centromere region (between dotted lines) plus its flanking sequences; (3) Composition of centromeric regions in terms of LTR-RTs, TRs, and other elements. (b) Phylogenetic tree constructed from all LTR-RTs identified in centromeric regions across species. Retrotransposon domains or subfamilies are color-coded, with branch colors indicating species identity. (c) Phylogenetic tree based on retrotransposon domains of CRs from the nine species. The outer circle indicates species, while the inner circle represents retrotransposon subfamilies.

Repeat annotations revealed that TRs and *Gypsy*-type LTR-RTs are the predominant components of centromeres, though their relative proportions vary among species. In *Arabidopsis* and pineapple, TRs account for more than 70% of the centromeric DNA, while *Gypsy* elements comprise less than 10% and 15%, respectively. Conversely, the centromeres of pepper and jujube are dominated by *Gypsy* elements, accounting for more than 50%, while TRs contributing less than 10% in both species. The remaining five species exhibit a more balanced distribution between TRs and *Gypsy* elements. Although centromeres across species display compositional diversities, CENH3 consistently defines the centromeric region through epigenetic level. To assess the evolutionary relationships among jujube and the eight other species, we constructed a phylogenetic tree based on their CENH3 amino acid sequences. The resulting topology is largely congruent with known species phylogenetic relationships, with monocots (pineapple, maize, and rice) clustering together and Rosid species (jujube, soybean, and cowpea) forming a separate clade (Fig. 4a).

Since LTR-RTs accounts for a large portion of centromere region in certain species, we extracted LTR-RTs from the centromeres of all nine species (including jujube) and classified them into 13 subfamilies based on domains as integrase, reverse transcriptase, and RNase H in LTR-RTs. Phylogenetic tree based on the retrotransposon domains of LTR-RTs revealed conservation of subfamilies across distant species. For instance, *Tekay*-type elements from pineapple, grape, and cowpea clustered together, despite their divergent lineages. Within each species, different subfamilies also showed distinct clustering; for example, the *Athila* and *CRM* elements from jujube were clearly separated into different clades (Fig. 4b).

To explore whether centromere-specific LTR-RTs like jujube CRJ are a universal phenomenon across species, we designed a metric called Centromeric Retrotransposon Enrichment Index (CRI) that can evaluate the centromere specificity of all centromere-located LTR-RTs. Based on the CRI, we identified 628 centromere-specific LTR-RTs across the nine species (Table S3). Genome-wide mapping confirmed that they are enriched in centromeric regions like CRJ in jujube. We call these 628 LTR-RTs as nCRs, representing nine species CRs. Species with a high proportion of *Gypsy* elements—such as jujube, pepper, and cowpea—exhibited stronger nCRs enrichment signals compared to species like *Arabidopsis* and pineapple, where *Gypsy* elements are less abundant (Fig. 4a).

To further investigate the evolutionary relationships among nCRs, we predicted domain structures for all 628 elements and found that 470 contained sufficient features for subfamily classification. A phylogenetic tree constructed using their transposon domain sequences revealed reduced subfamily diversity compared to the total centromeric LTR-RTs as shown in Fig. 4b, with most nCRs falling into three major subfamilies: *CRM*, *Athila*, and *Tekay*. Among them, *CRM* was the most abundant, comprising 81.2% (398 elements), followed by *Tekay* (9.7%, 48 elements) and *Athila* (8.5%, 42 elements). The remaining two subfamilies were represented by only one element each. Notably, nCRs from different species clustered by subfamily rather than species. For example, 99% of CRJ belonge to the *Athila* and *CRM* subfamilies—*Athila*-type CRs clustered closely with those *Athilas* from *Arabidopsis*, while *CRM*-type CRs grouped with those *CRMs* from soybean and cowpea. A single *Ivana*-type CR from jujube clustered with maize *CRM* elements. All CRs of pineapple and grape were classified as *Tekay*-type and formed a distinct clade (Fig. 4c).

### Expansion of centromeric LTRs contributes to the centromere TRs formation

**Figure 5:**
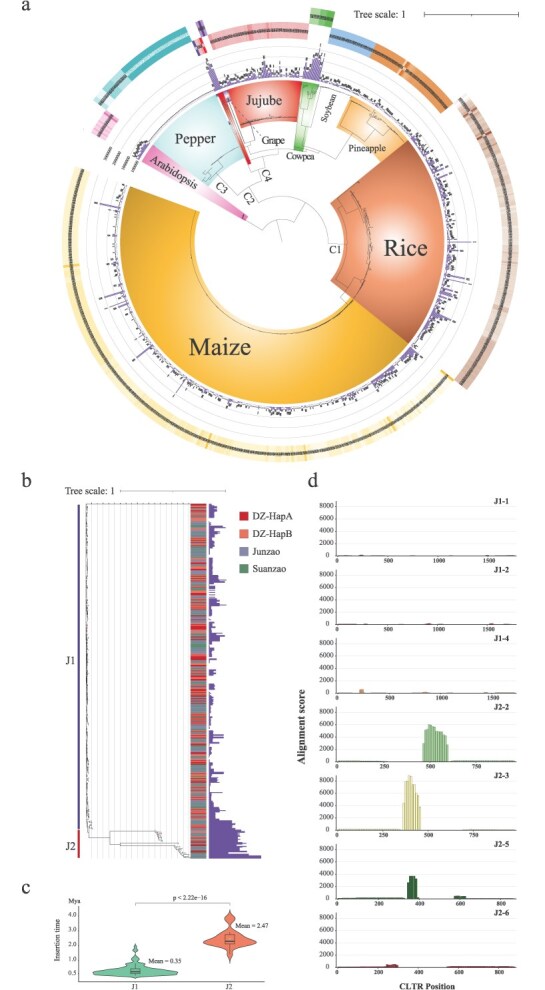
Evolutionary dynamics of centromeres inferred from CLTRs. (a) Circular phylogenetic tree constructed using CLTR sequences from nine species. The outermost circle displays a heatmap of CLTRI values for each species, while the inner bar plot circle illustrates the LTR insertion times. (b) Phylogenetic tree based on CLTRs from three jujube individuals. The stacked bar plots on the left show the distribution of CLTRs among the three genotypes, while the bar plots on the right depict insertion times. Two major clades are annotated as J1 and J2. (c) Comparative analysis of insertion times between J1 and J2 clades. Violin plots combined with boxplots illustrate the distribution of insertion times, with the p-value indicating statistical significance. (d) Correlation between CLTRs and centromeric TRs in J1 and J2. The *x*-axis shows 10-bp bins across CLTR regions, and the *y*-axis represents alignment scores, indicating the degree of similarity between CLTRs and TRs.

Centromeric LTRs (CLTRs), as essential structural components of LTR-RTs, play pivotal roles in both targeted genomic insertion and expansion. As demonstrated in jujube (Fig. 2b), the expansion of CRJ primarily originates from their flanking LTR regions. To evaluate the generality of this pattern, we extracted the CLTRs from the nCRs of the above eight species and constructed a phylogenetic tree based their sequences. The resulting tree revealed that *Arabidopsis* CLTRs diverged earliest, followed by the emergence of two major clades, designated C1 and C2. C1 comprised the CLTRs from maize and rice, while C2 encompassed the remaining species. Within C2, two divergent CLTRs from soybean were the first to branch off, followed by the formation of two subclades: C3, containing CLTRs from pepper, and C4, comprising the CLTRs of the remaining species. In C4, jujube and grape clustered together, whereas cowpea, pineapple, and the remaining soybean CLTRs formed a separate cluster. Notably, the monocot pineapple did not cluster with rice and maize, but instead grouped with dicot species, suggesting a lineage-specific divergence in its CLTR elements (Fig. 5a). The lengths of CLTRs within the same species typically exhibited a consistent distribution pattern. For instance, jujube CLTRs predominantly fell into two size classes—approximately 1700 and 800 bp—while those from pepper were centered around 750 bp, and those from maize around 900 bp (Fig. S9). Moreover, CLTRs from the same species tended to cluster together, forming distinct species-specific lineages with sequence similarities exceeding 80%.

To investigate the evolutionary patterns of CLTR insertion, we calculated the centromeric LTR Retrotransposon Enrichment Index (CLTRI), which quantifies the relative enrichment of CLTRs in centromeric regions compared to noncentromeric regions. In jujube, two major CLTR subclades were identified, separated by grape CLTRs. The right subclade, which contained the majority of CLTRs, exhibited relatively stable CLTRI values ranging from 2.44 to 3.35. In contrast, the left subclade, comprising fewer CLTRs, included a pair with CLTRI values exceeding 5.1, while the remainder ranged from 2.34 to 3.16. Additionally, CLTRs in the right subclade were more recently inserted than those in the left subclade. In pepper, all CLTRs exhibited very recent insertion times, and could thus be considered evolutionary young. Across its phylogenetic clades, CLTRI values gradually increased from left to right. In cowpea, two distinct clades showed clear differences in CLTRI, with insertion time positively correlated with enrichment—older CLTRs tended to have higher CLTRI values (Fig. 5a). CLTRI reflects the degree of centromeric localization and these findings suggest that older CLTRs may have undergone greater copy number expansion within centromeres in evolution.

To further dissect the evolutionary process of jujube centromere, we incorporated previously published T2T genome of two other individuals, ‘Junzao’ (JZ) and ‘Suanzao’ (SZ) [25], identified genome-wide LTR-RTs in each species and screened their CLTRs using CLTRs of DZ, and then constructed the phylogenetic tree using CLTRs from these three individuals (Fig. 5b). The two major subclades of jujube in Fig. 5a were clearly displayed here and designated them as J1 and J2. The J1 clade had a significantly more recent insertion time of 0.35 Mya than the 2.46 Mya in J2 clade (*P* < 2.22 × 10^−16^, Wilcoxon test, Fig. 5c). Notably, as shown in Fig. 4c, the majority of CRJs assigned to the *Athila* subfamily belonged to the J1 group, whereas the minority of CRJs associated with the *CRM* and *Ivana* subfamilies were primarily clustered within the J2 group.

Subsequently, CD-HIT was employed to cluster CLTRs from the two subclades based on >70% sequence identity, reducing the 417 CLTRs in the J1 subclade to four representative sequences (J1-1 to J1-4), and the 37 CLTRs in the J2 subclade to six (J2-1 to J2-6). Meanwhile, approximately 1.08 Mb bp centromere TR sequences (CTRs) were identified within three jujube genomes using trf (v4.10.0), accounting for 2.62% of the total centromeric sequence. The ten representative CLTRs were aligned to these CTRs, yielding alignments spanning 127 564 bp of CTRs. Alignment scores were then calculated in 10 bp bins to examine their distribution across the CTR regions. The results revealed that J1-3, J2-1, and J2-4 failed to align with any CTR sequences, while J1-1, J1-2, J1-4, and J2-6 exhibited only weak alignment signals and were excluded from further consideration. In contrast, J2-2, J2-3, and J2-5 displayed strong alignment signals, each containing a distinct high-scoring interval. These intervals measured 140 bp for J2-2, 100 bp for J2-3, and 50 bp for J2-5, with average alignment scores of 4954, 8140, and 4392, respectively. Notably, the 140 bp interval in J2-2 shares 88.66% sequence similarity with the 100 bp interval in J2-3 and 95.35% similarity with the 50 bp interval in J2-5, suggesting that these regions are homologous (Fig. 5d). Collectively, the high-scoring alignment intervals from J2-2, J2-3, and J2-5 covered 120 508 bp, accounting for 94.47% of the total aligned CTR sequences. Furthermore, collinearity analysis for J2-2, J2-3, and J2-5 clearly demonstrated the evolutionary transition of CLTRs into TRs (Fig. S10). These findings suggest that older CLTRs may undergo sequence homogenization and localized expansion, progressively evolving into CTRs during evolution.

## Discussion

Centromeres are fundamental for ensuring accurate chromosome segregation during cell division and for maintaining genome stability in plants. This critical function is primarily mediated through the recruitment of CENH3 and the assembly of kinetochores [6]. Recent advances in sequencing technologies have greatly accelerated the development of T2T genome assemblies, enabling the precise identification and characterization of centromeres across a wide range of species [26]. Furthermore, plant centromeres were traditionally thought to be predominantly composed of TR sequences, as exemplified in *Arabidopsis* [8], sugarcane [27], and Chinese cabbage [10]. However, recent studies have revealed that LTR-RTs also serve as a major structural component of centromeres in certain plant species, such as pepper [15] and tobacco [16]. Despite these advances, detailed investigations into the centromere organization of fruit trees remain limited to date.

Previously, our T2T assembly of DZ jujube revealed an unconventional centromere structure characterized by the absence of extensive TRs [23]. Based on this, we further assembled a haplotype-resolved T2T assembly of DZ and precisely delineated centromere boundaries across chromosomes using CENH3 ChIP-seq experiments. RNA-seq data revealed that jujube centromeres exhibit hallmark features commonly observed in model plants such as *Arabidopsis* and tobacco, including low gene density and globally reduced gene expression [8, 16]. DNA methylation in centromeres remained largely unchanged, except on chromosome 1, which exhibited a marked decrease in its central region. This aligns with previous findings that, although centromeres are typically heavily methylated, regions like CENH3-binding sites or LTRs often exhibit lower methylation to preserve centromere function [6]. Unlike traditional centromeres, which are typically dominated by TRs spanning several hundred kilobases to megabases, jujube centromeres are primarily composed of LTR-RTs, particularly those of the *Gypsy* family, which accounts for 54.52% of the centromeric regions. This composition closely parallels that observed in Solanaceae species such as pepper [15] and potato [28], where LTR-RTs similarly dominate centromeric regions, comprising 59.55% and 49.20%, respectively.

Further analysis identified a specific group of LTR-RTs predominantly enriched within the jujube centromeres, which we designated as CRJs. Both homology-based analyses and CENH3 ChIP-seq mapping confirmed that CRJ elements are more enriched in centromeric regions compared to non-CRJ LTR-RTs. Additionally, the two terminal LTR regions of CRJs showed stronger enrichment than their internal domains, indicating a closer association between LTR sequences and centromeric chromatin. Based on this observation, we designed FISH probes using sequences from the LTR region of CRJs to validate centromere localization. This approach, using LTR-derived sequences as probes, has been reported in other species such as maize [29], rye [30], and cucumber [31], but has not been applied to pepper [15] and tobacco [16], which exhibit similar centromere organization to that of jujube. Although we successfully colocalized the centromeres on all chromosomes, the FISH signals were relatively weak, likely due to the limited copy number of CRJ elements. FISH signal intensity is closely related to the abundance of the probe target sequence [32]. In HapA, for example, the estimated maximum and minimum copy numbers of the 376 bp fragment were 28 and 11, respectively (Table 2), and this relatively low abundance limits the probe’s ability to robustly label the centromeres, in contrast to species like *Arabidopsis*, where centromeres are primarily composed of highly abundant TRs [8]. Despite this limitation, our successful application of using probes derived from the LTR region of CRJ provides the first proof of concept for centromere localization in species whose centromeres are dominated in LTR-RTs but lack canonical TRs.

Comparative analyses of nine plant species, including jujube, with ChIP-seq-validated centromeres revealed three primary modes of centromere organization: those dominated by TRs (e.g. *Arabidopsis*), those dominated by LTR-RTs (e.g. jujube and pepper), and those where TRs and LTR-RTs coexist (e.g. soybean, cowpea, grape, pineapple, maize, and rice) (Fig. 4a; Table S4). Across these species, LTR-RTs in centromeric regions were classified into 13 subfamilies (Fig. 4b). Notably, a subset of these, nCRs, clustered into three major subfamilies: *CRM*, *Tekay*, and *Athila* (Fig. 4c). This clustering pattern suggests structural conservation among CRs and implies the presence of evolutionary constraints that favor specific subfamilies during centromere evolution. Additionally, comparative analysis of CLTRs revealed over 80% sequence similarity across the nine species, indicating a common evolutionary origin. However, the phylogenetic tree based on CLTR sequences did not mirror species relationships; for instance, the monocot pineapple grouped within the dicot lineage (Fig. 5a). This phylogenetic incongruence may point to frequent horizontal exchange or convergent evolution of CLTR sequences.

Although jujube and pepper shared the most similar centromeric architecture, the insertion times of their CLTRs differed markedly. In pepper, CLTRs predominantly represent recent insertions, consistent with previous studies [15]. In contrast, jujube centromeres show evidence of both ancient and more recent CLTR insertion bursts, highlighting a more complex evolutionary history. To further explore this, we analyzed two major CLTR subgroups in jujube, designated J1 and J2. A 100-bp internal fragment within the J2 subgroup showed high sequence similarity to CTRs found outside of LTR-RTs. Although CTRs account for only ~3.3% of the jujube centromere, over 10% of these CTRs could be traced to this J2-derived fragment. This provides compelling evidence that some TRs may originate from degenerated CLTR sequences. Supporting this, the J2 subgroup exhibits greater structural degradation and shorter element length compared to J1, suggesting a possible transition from retrotransposon to TR through progressive sequence erosion. This pattern aligns well with recently findings in other plant species. For example, in tobacco, both satellite-enriched and satellite-free centromeres are extensively invaded by distinct *Gypsy*-type retrotransposons [16]. In maize, CTR clusters *CRM1TR* and *CRM4TR* share 97% sequence identity with *CRM* retrotransposons [19], supporting a retroelement-to-repeat conversion pathway. In *Solanum* species, centromeres have been shown to evolve rapidly from neocentromeres (regions lacking typical tandem repeats) through *de novo* amplification and undergo boom-bust cycles before a favorable TR is fixed [20, 21]. Similarly, a recent study in *Arabidopsis* reported rapid turnover of both *Ty3*- and *Ty1*-type LTR-RTs within centromeric TRs, offering further support for our observations [33].

Building on these observations, we propose a model for centromere evolution in jujube characterized by periodic bursts of CRJ insertions that drive centromeric expansion, followed by sequence fragmentation and eventual stabilization. These episodic insertion events gave rise to CLTRs of varying lengths, with older J2 elements (~750 bp) and younger J1 elements (~1700 bp) (Figs 5c and S9). Over time, some ancient CLTRs likely underwent further expansion and degeneration, ultimately giving rise to the shorter CTRs now observed. This dynamic turnover underscores the pivotal role of LTR-RT proliferation in reshaping centromere architecture during the chromosomal evolution of jujube. Given the essential function of centromeres in ensuring accurate chromosome segregation, such as large-scale restructuring, via LTR insertions, degeneration into CTRs, and subsequent epigenetic reorganization, could disrupt homologous chromosome pairing during meiosis. These structural and epigenetic shifts may have contributed to reproductive isolation, supporting a potential role for centromere evolution in speciation. This hypothesis aligns with the ‘genomic shock’ model, which posits that bursts of transposon activity destabilize genome integrity and act as a driver of evolutionary divergence [34–36]. Intriguingly, the estimated timing of major CLTR insertion bursts in jujube coincides with pronounced climatic oscillations during the early Pleistocene (~2.58–0.78 million years ago). This temporal overlap suggests that environmental stress may have triggered transposable element activation, thereby accelerating centromere remodeling and potentially influencing broader genome evolution.

## Conclusion

In conclusion, this study provides the first comprehensive characterization of centromere structure in jujube based on haplotype-resolved T2T assemblies and functional CENH3 ChIP-seq validation. We reveal that jujube centromeres are primarily composed of lineage-specific LTR-RTs rather than TRs, and demonstrate that ancient centromere-specific LTRs have contributed to the emergence of satellite DNA. These findings advance our understanding of centromere evolution in perennial woody plants and highlight the dynamic role of transposable elements in shaping chromosome architecture and genome stability. Nevertheless, certain limitations still remain in current research, including the reliance on a single cultivar and the absence of direct evidence for centromere activity beyond CENH3 localization, such as functional assays or cytogenetic localization, which will be addressed in future studies. Overall, our study expands current knowledge of centromere organization in fruit trees and provides a high-quality genomic resource for future research in chromosome biology, genome evolution, and molecular breeding in Rhamnaceae and beyond.

## Materials and methods

### Haplotype assembly, annotation, and comparative genomics

The HiFi ccs reads, passed ultra-long ONT reads, and Hi-C sequencing data were used as input for Hifiasm software (v0.20.0-r639) [37] with the parameter ‘--ul-cut 15000 -D10 --hom-cov 71’. Preliminary haplotype-resolved assemblies were directly obtained in the output files with the suffixes ‘hap1.p_ctg.gfa’ and ‘hap2.p_ctg.gfa’. Following the removal of organelle-derived sequences, Purge_dups [38] was employed to eliminate redundant, low-coverage, and artifactual contigs. The remaining contigs were aligned to our previous published T2T assembly of DZ using mummer (v4.0.0rc1) [38] to refine orientation and determine the final haplotype-resolved assemblies (HapA and HapB).

Genome annotation was performed following our previously established pipeline [23]. Briefly, repetitive elements were identified primarily using RepeatMasker. Intact LTR-RTs were detected using LTR_retriever (v2.9.0) [39], and their classification into subfamilies was performed with TEsorter (v1.4.6) [40] based on the rexdb-plant database, which enabled the identification of key retrotransposon domains in LTR-RTs including integrase (INT), reverse transcriptase (RT), and ribonuclease H (RH). Protein-coding gene annotation was conducted through an integrated approach combining protein homology, transcriptomic evidence, and *ab initio* gene prediction. The completeness of both haplotype assemblies and their corresponding protein-coding gene annotations was evaluated using the embryophyta_odb10 dataset within the BUSCO (v5.8.2) [41]. The collinear regions within each haplotype were independently identified using MCScanX [42] with default parameters. To compare the two haplotypes (HapA and HapB), genome-wide alignment was performed using MUMmer (v4.0.0rc1), followed by visualization with GenomSyn (v1.2.7) [43]. Circular plots illustrating synteny relationships were generated using Circos (v0.69–8) [44].

### Identification of centromeres

To locate the centromere position of jujube, ChIP-seq was performed using a rabbit polyclonal anti-CENH3 antibody raised against the synthetic peptide LAERKLRRPSGGVSTPSPRK, as described in *Forsythia suspensa* [45]. The antibody was produced and supplied by AtaGenix Laboratories Co., Ltd. (Wuhan, PR China). Approximately, 5 g of jujube seedling tissue derived from *in vitro* culture was harvested, washed twice with deionized water, and cross-linked with 1% formaldehyde under vacuum for 10 min. The cross-linking reaction was quenched by adding glycine to a final concentration of 125 mM. The samples were then lysed on ice to release chromatin, which was subsequently sonicated to obtain DNA fragments with an average length of 200–500 bp. For each ChIP assay, 100 μl of chromatin was used for immunoprecipitation with the anti-CENH3 antibody, while 20 μl of chromatin was retained as input control. The immunoprecipitated DNA was used to construct 150 bp paired-end sequencing libraries following the manufacturer’s protocol, and the library was sequenced on the Illumina NovaSeq 6000 platform by Wuhan Biorun Biosciences Co., Ltd.

Raw paired-end reads were first preprocessed using fastp (v0.23.2) [46] to trim low-quality bases and remove adapter contamination. The quality-controlled reads were then independently aligned to the HapA and HapB genome assemblies using Bowtie2 (v2.5.1) [47] with default parameters. Alignments with a mapping quality score below 20 were filtered out using Samtools (v1.15) [48]. To identify centromeric regions enriched for CENH3 binding, the bamCompare tool from the DeepTools package (v3.5.1) [49] was used to generate genome-wide enrichment profiles by calculating the signal ratio between ChIP-seq and input control data. The resulting enrichment signals were computed over 1 kb genomic bins and converted into bedGraph format. The putative centromeric regions for each chromosome were manually examined and refined using the Integrative Genomics Viewer (IGV, v2.18.4) [50], thereby enabling the accurate delineation of centromere boundaries. The distribution of aligned ChIP-seq reads across chromosomes was visualized using pyGenomeTracks (v3.9) [51].

### Identification of CRJs and visualization

Repetitive sequences identified through whole-genome annotation using RepeatMasker were first extracted from the centromere regions. These sequences encompassed various repeat families, and those families with high copy number in the centromere were selected as representative candidates. These families were then aligned to the HapA genome, and their distribution was visualized using IGV [50]. Repeat families predominantly localized within centromeric regions were retained as centromere-associated candidates. Subsequently, intact LTR-RTs within the centromeres were identified using LTR_retriever [39]. The candidate repeat sequences were aligned against all intact LTR-RTs from the centromeric regions to identify the most frequently occurring elements. These were defined as the CRJs. Among the CRJs, the element with a sequence length most similar to the initial candidate was selected as the representative CRJ and is referred to as CRJ in this study. The CRJ sequence was subsequently aligned against the entire genome, and alignment scores for each 100 bp bin of CRJ were calculated using the formula:


\begin{equation*}{S = L \times N}\end{equation*}

where *S* represents the alignment score, *L* is the alignment length per 100 bp bin of the CRJ sequence, and *N* is the number of alignment hits for each 100 bp bin. These alignment scores were visualized to assess the genome-wide distribution and repetitive frequency for different region of CRJ sequence. The retrotransposon domains within CRJ, previously annotated using TEsorter, were visualized using IBS (v2.06) [52]. To assess whether CRJ elements are enriched in centromeric regions at the raw sequencing level, we aligned both CRJ and a non-CRJ control (an LTR-RT randomly selected from centromere region) to the reference genome. The ChIP-seq enrichment signal was calculated as the ratio of ChIP-seq reads to Input reads (ChIP/Input) at the corresponding alignment sites. Enrichment patterns for CRJ and non-CRJ elements were then analyzed using the computeMatrix tool from the deepTools package (v3.5.1).

### FISH experiment for jujube centromere

Fluorescence *in situ* hybridization (FISH) was performed as described by Huang *et al.* [53]. Jujube seeds were germinated in a growth chamber at 30°C. After 2–3 days, roots (1–2 cm in length) were harvested and treated with 1 MPa N₂O for 30 min, followed by fixation in Carnoy’s solution (ethanol:glacial acetic acid = 3:1) at −20°C overnight. Fixed roots were rinsed three times with ddH₂O (5 min per wash), and only the root tips were subjected to enzymatic digestion (2% cellulase and 1% pectinase at 37°C for 2 h). The digested root tips were then squashed onto poly-L-lysine-coated slides.

Three 5′-TARMA-labeled oligonucleotide probes, CRJ-1-TARMA, CRJ-2-TARMA, and CRJ-3-TARMA, were designed from a 376-bp region of the CRJ retrotransposon. These probes target distinct regions of the sequence and were each directly labeled with TARMA, a red fluorophore (Fig. S5). To enhance signal intensity and coverage, the probes were pooled for hybridization. To conduct FISH, chromosomal DNA was denatured in 70% formamide/2× SSC (saline sodium citrate) at 82°C for 2 min, followed by sequential dehydration with 70%, 95%, and 100% ethanol. And then incubated with probes mixture at 37°C for overnight. Slides were washed with 2× SSC for four times, twice at room temperature and twice at 42°C. Nuclei were counterstained with DAPI, and fluorescence signals were visualized using an Olympus BX61 fluorescence microscope equipped with a CCD camera (QImaging; RETGA-SRV FAST 1394). Digital images were analyzed using Image-Pro Plus 6.0 (Media Cybernetics). Three biological replicates were performed to ensure reproducibility and reliability of the results.

### Multiple species CRs/CLTRs identification and evaluation

Genome sequences from eight species with experimentally validated centromeres based on CENH3 ChIP-seq data were downloaded. These species include *V. vinifera* [54], *A. thaliana* [8], *C. annuum* [15], *A. comosus* [55], *Z. mays* [56], *O. sativa* [57], *V. unguiculata* [58], and *G. max* [59]. For each species, the centromeric regions were extracted as previously described, followed by identification of LTR-RTs.

To identify CRs form the centromere LTR-RTs, we developed a metric termed the Centromeric Retrotransposon Enrichment Index (CRI), calculated as follows:


$$ \mathrm{CRI}=\frac{\sum \mathrm{CEN}\left({L}_i\ast{I}_i\right)}{\sum \mathrm{Non}-\mathrm{CEN}\left({L}_j\ast{I}_j\right)} $$

where CRI represents the CR enrichment index; CEN and non-CEN indicate alignments within centromeric and noncentromeric regions, respectively; *L_i_* denotes the alignment length of the *i*-th alignment of a CR sequence to the centromeric region, and *I_i_* represents the corresponding alignment identity. Likewise, *L_j_* and *I_j_* represent the alignment length and identity of CR alignments to noncentromeric regions.

For CLTRs, we extracted the LTR sequences flanking the identified CRs and CLTRI using the same formula described above.

Using transposon regions and the CLTRs of CRs from the nine species (including jujube), we respectively conducted multiple sequence alignments and constructed phylogenetic trees using iqtree (v2.2.0) [60]. Finally, we visualized the phylogenies using [61] integrating both the estimated insertion times of LTRs and their corresponding enrichment index.

### Statistical statements

Wilcoxon rank-sum test was used in the one set of data statistics in Fig. 5c.

## Supplementary Material

Web_Material_uhaf244

## Data Availability

The raw data of ChIP-seq are deposited in the National Genomics Data Center with BioProject: PRJCA039671. The haplotype-resolved genome and annotation file have been shared in the figshare repository at https://figshare.com/s/e040dc92e6ceb9ec232d.
