# Supplementary material for: Structural composition and evolution of jujube centromere reveal a dominant role for LTR retrotransposon
Source: Hortic Res. 2025 Sep 15;12(11):uhaf244. doi: 10.1093/hr/uhaf244 (PMC12596083; doi:10.1093/hr/uhaf244)
Supplement: Web_Material_uhaf244 [file web_material_uhaf244.zip › Supplementary_Figures.docx]

Supplementary Figures


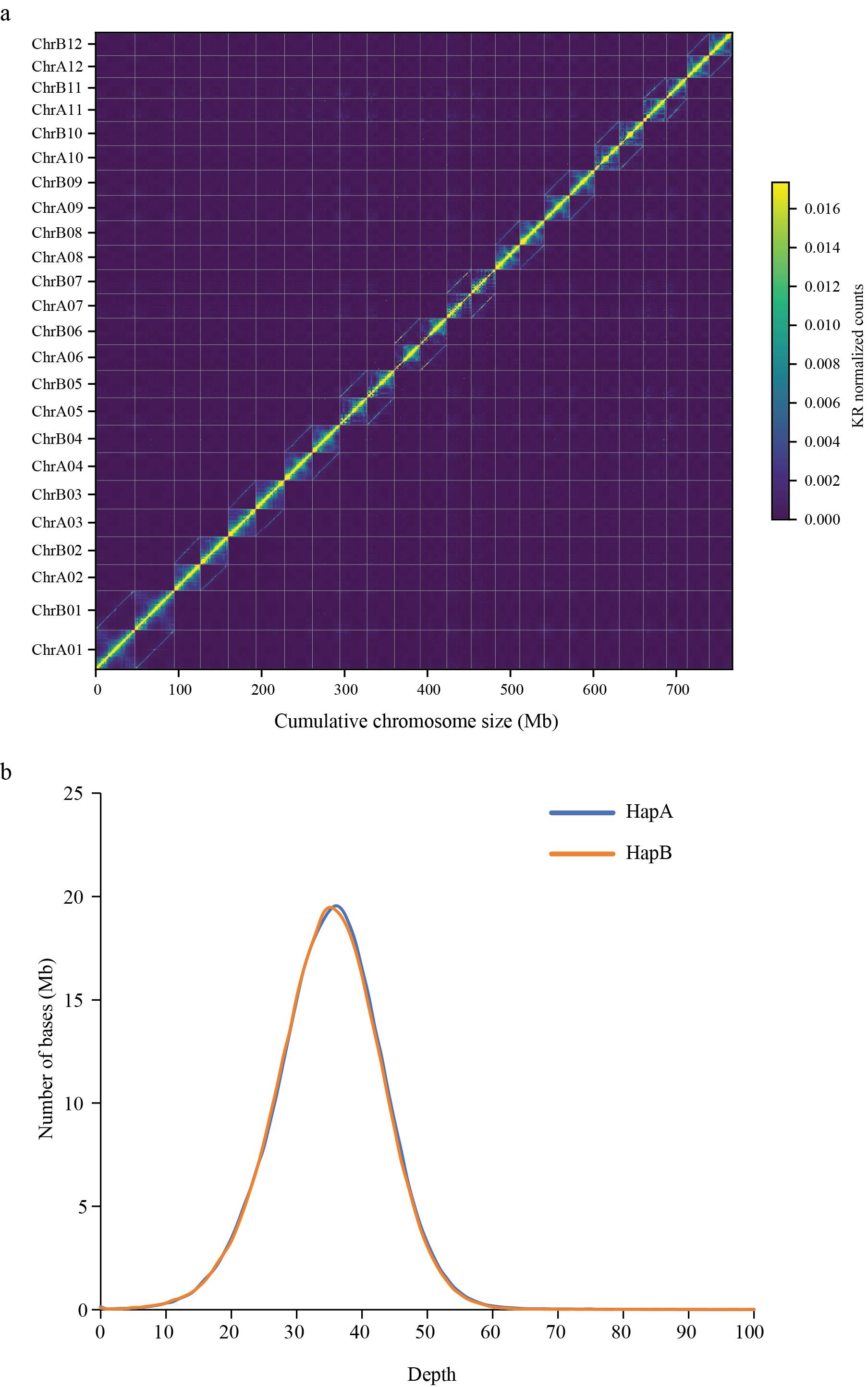


Figure S1. Quality assessment of the haplotype-resolved assembly of the DZ genome. a. Hi-C contact map at 100 kb resolution generated using HapHIC. Contact intensities are normalized using the Knight-Ruiz (KR) method. b. HiFi read depth distribution across both haplotypes.


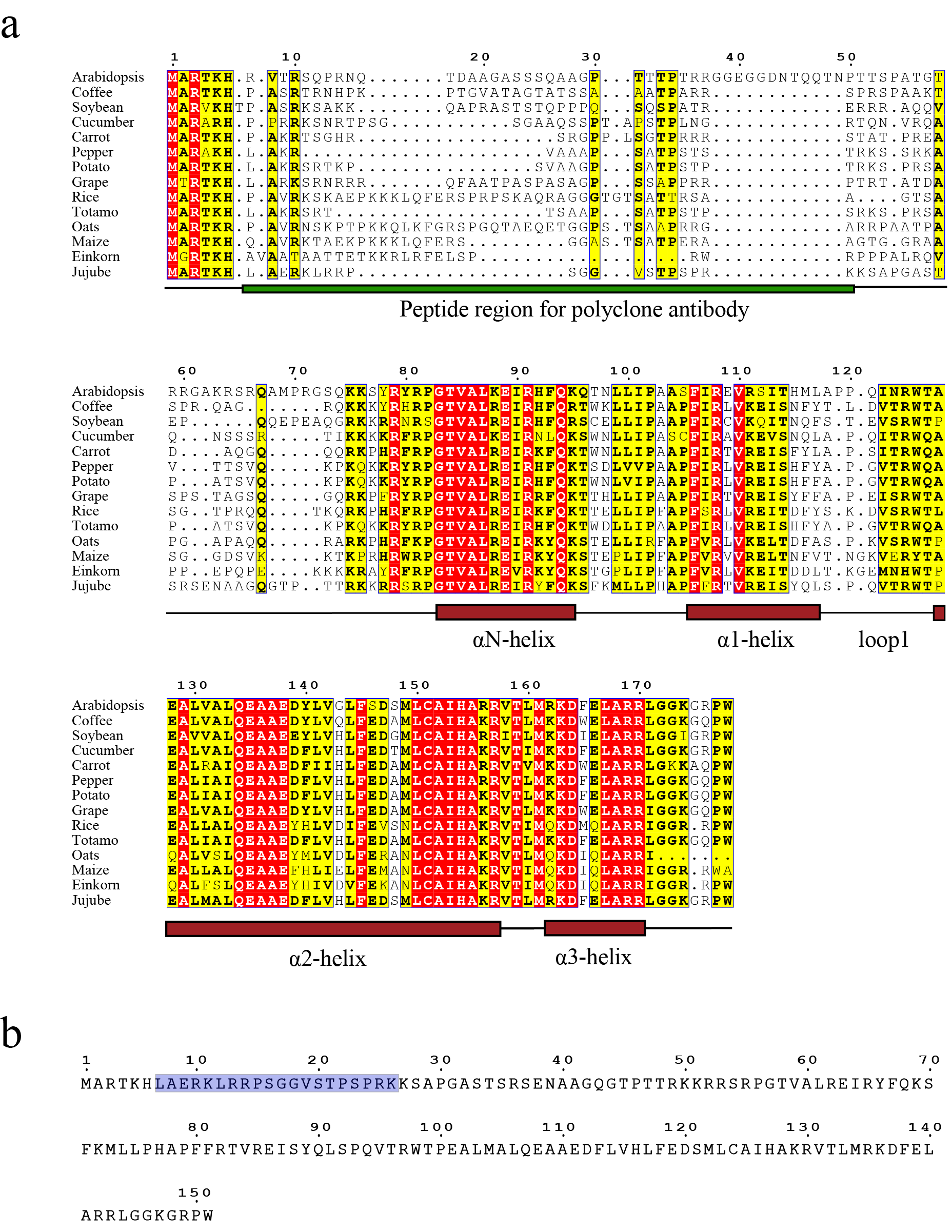


Figure S2. Multiple sequence alignment and structural domain annotation of CENH3 proteins from 13 plant species. a. Multiple sequence alignment of CENH3 proteins with structural domains highlighted in brown-red ribbons. The peptide used for producing the polyclonal antibody is indicated by a green ribbon. b. The 151-amino-acid sequence of Jujube CENH3, with the polyclonal antibody target peptide highlighted in blue.


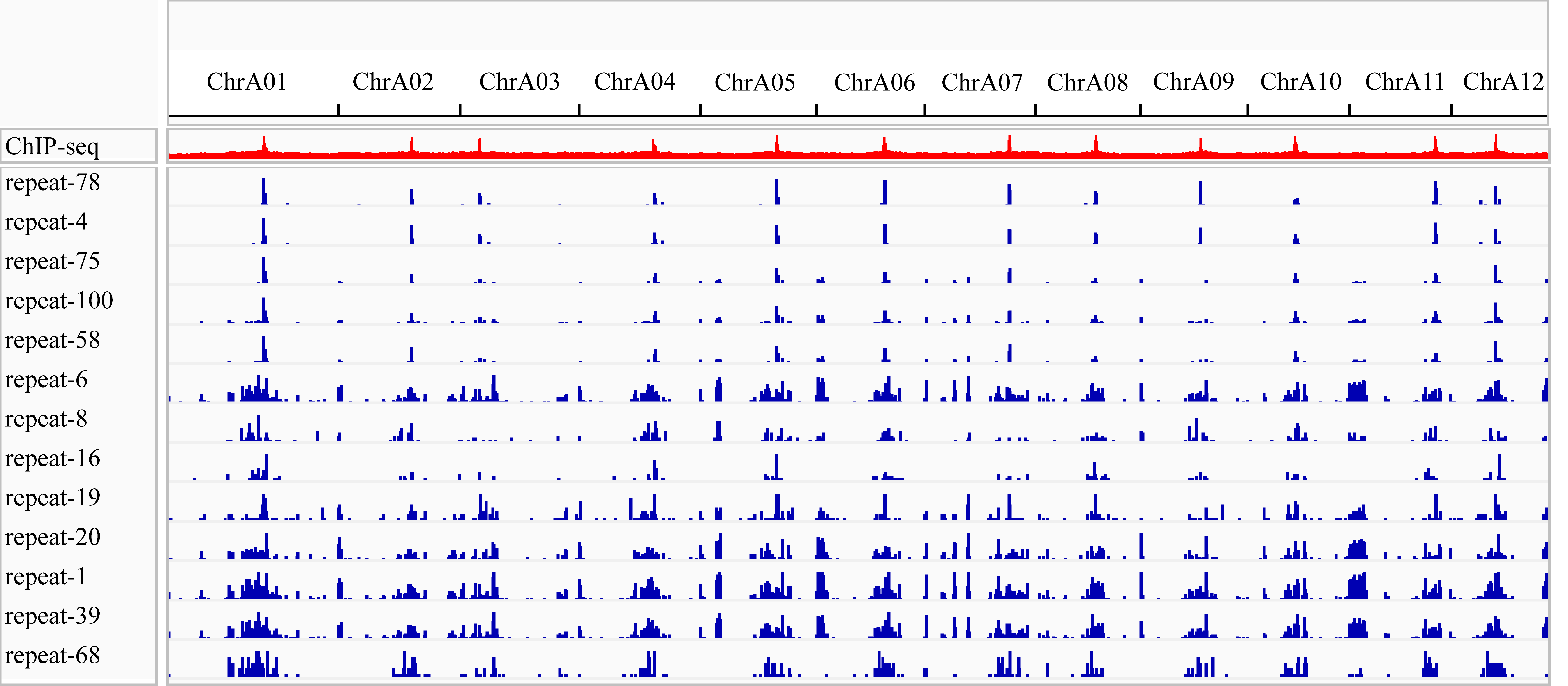


Figure S3. Genome-wide distribution of different repeat families in centromeric regions identified by RepeatMasker. Repeat families detected in the centromeric regions by RepeatMasker were aligned to the whole genome, and the alignment results are displayed. The top red panel shows ChIP-seq peak signals, while the blue panels below represent the genome-wide alignments of each repeat family corresponding to the centromeric repeats above.





Figure S4. Genome-wide distribution of CRJ elements. Each chromosome is represented by two panels: the upper gray panel shows the distribution of ChIP-seq reads, while the lower panel displays the genome-wide alignment of CRJ elements. In the lower panel, red indicates the positions of the two terminal LTRs, and blue indicates the internal retrotransposon domains. Both panels use a 100 kb window bin for visualization.


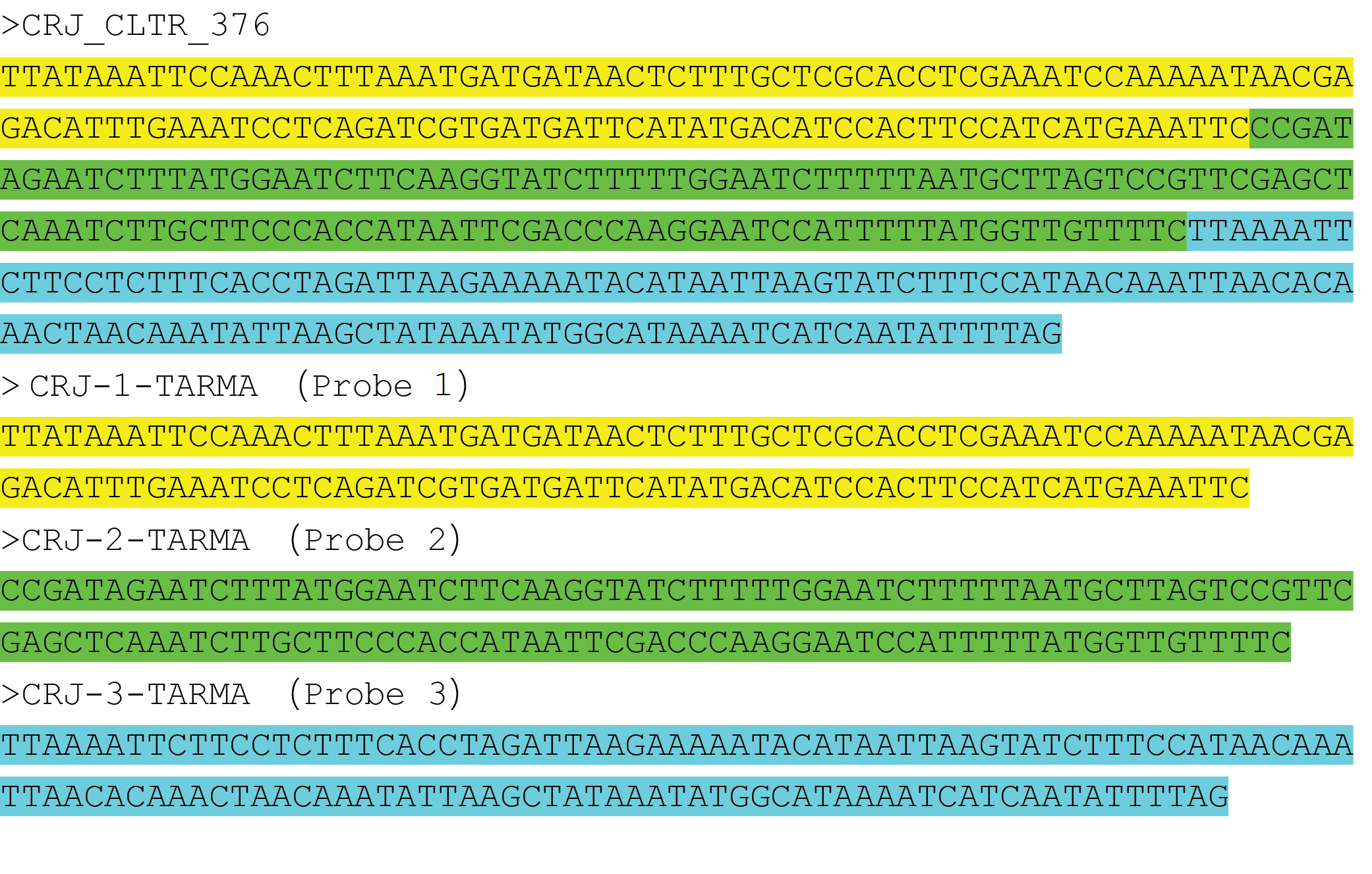


Figure S5. Sequences of probes used for the FISH experiment. Three 5’-TARMA-labeled oligonucleotide probes, CRJ-1-TARMA, CRJ-2-TARMA, and CRJ-3-TARMA, are highlighted in yellow, green, and cyan, respectively.


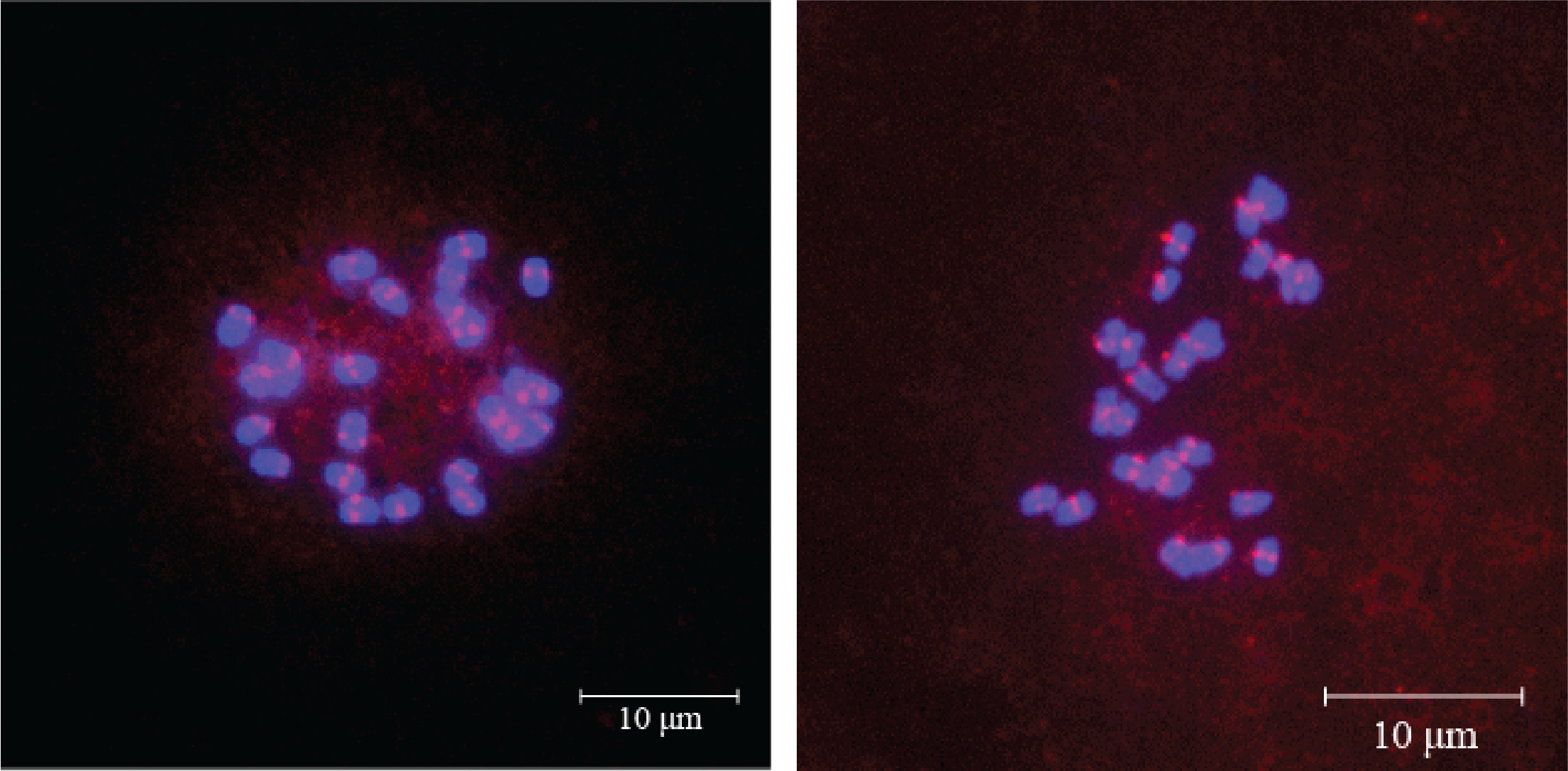


Figure S6. Two additional replicates of FISH results using the three CRJ-specific probes.


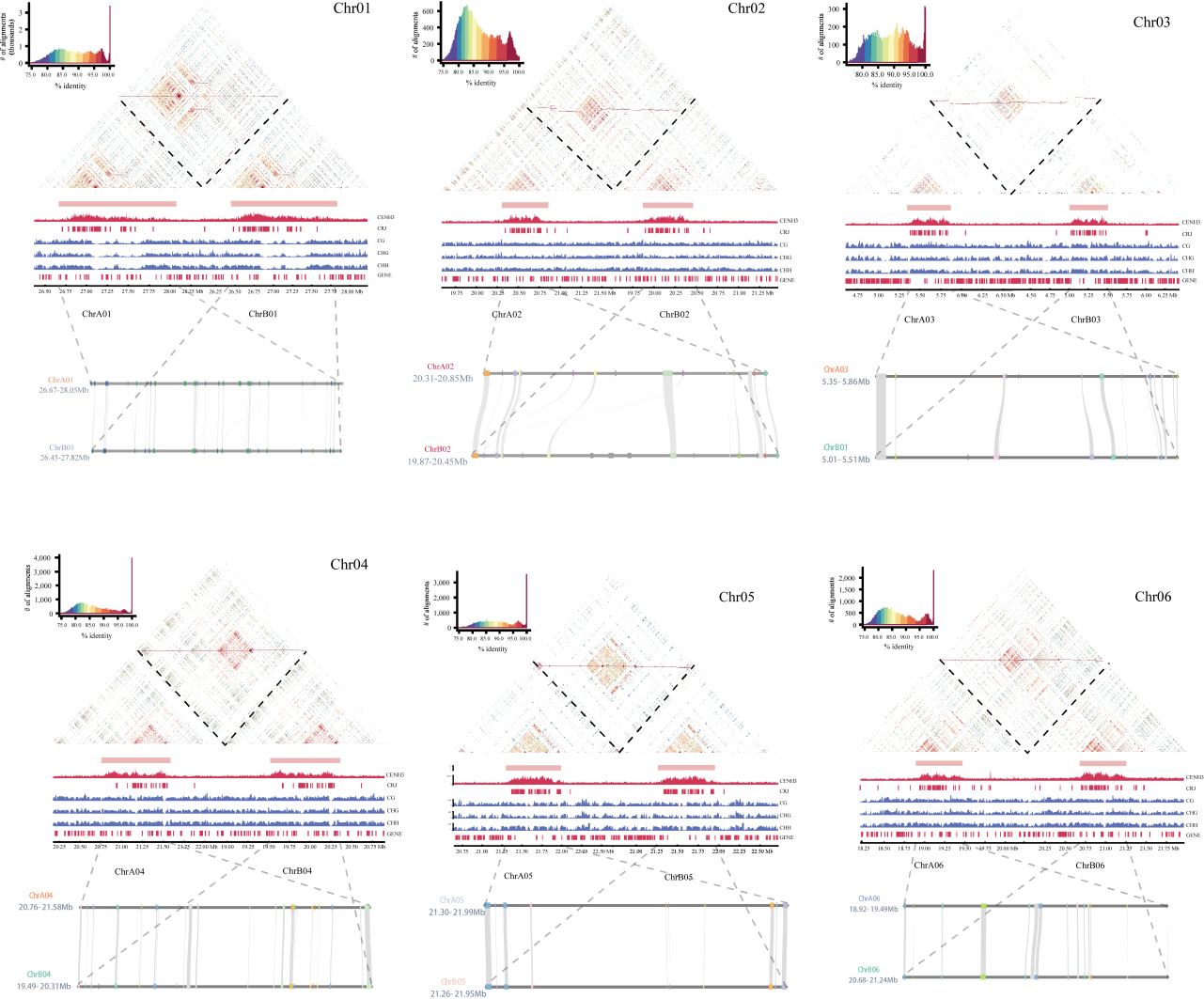


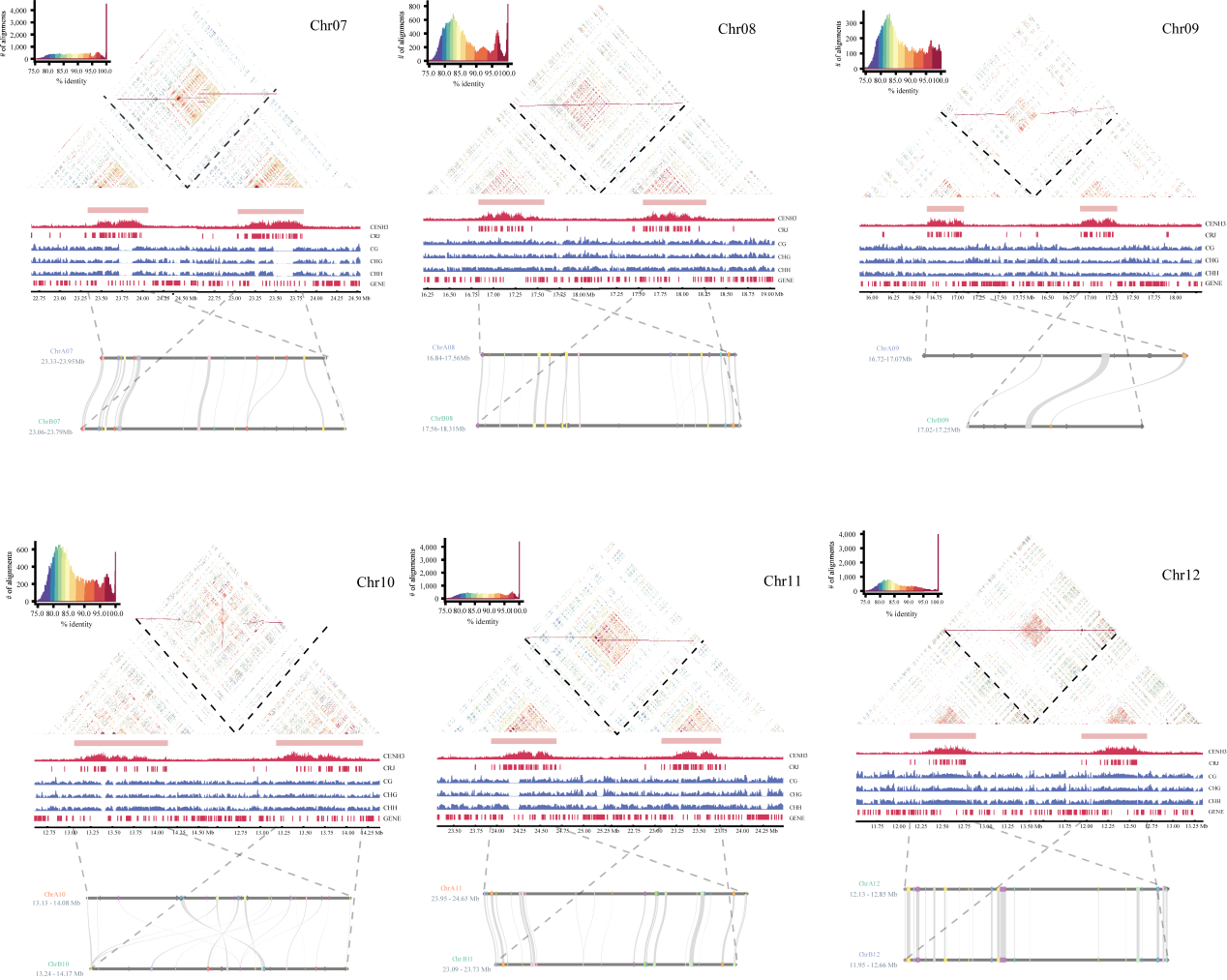


Figure S7. Epigenetic features of 12 chromosomes comparing HapA and HapB. From top to bottom: sequence similarity matrix between HapA and HapB, intra-haplotype similarity matrices for HapA and HapB, CENH3 ChIP-seq peaks, CRJ alignment positions, DNA methylation levels in CG, CHG, and CHH contexts, gene density, and gene collinearity between the two haplotypes.


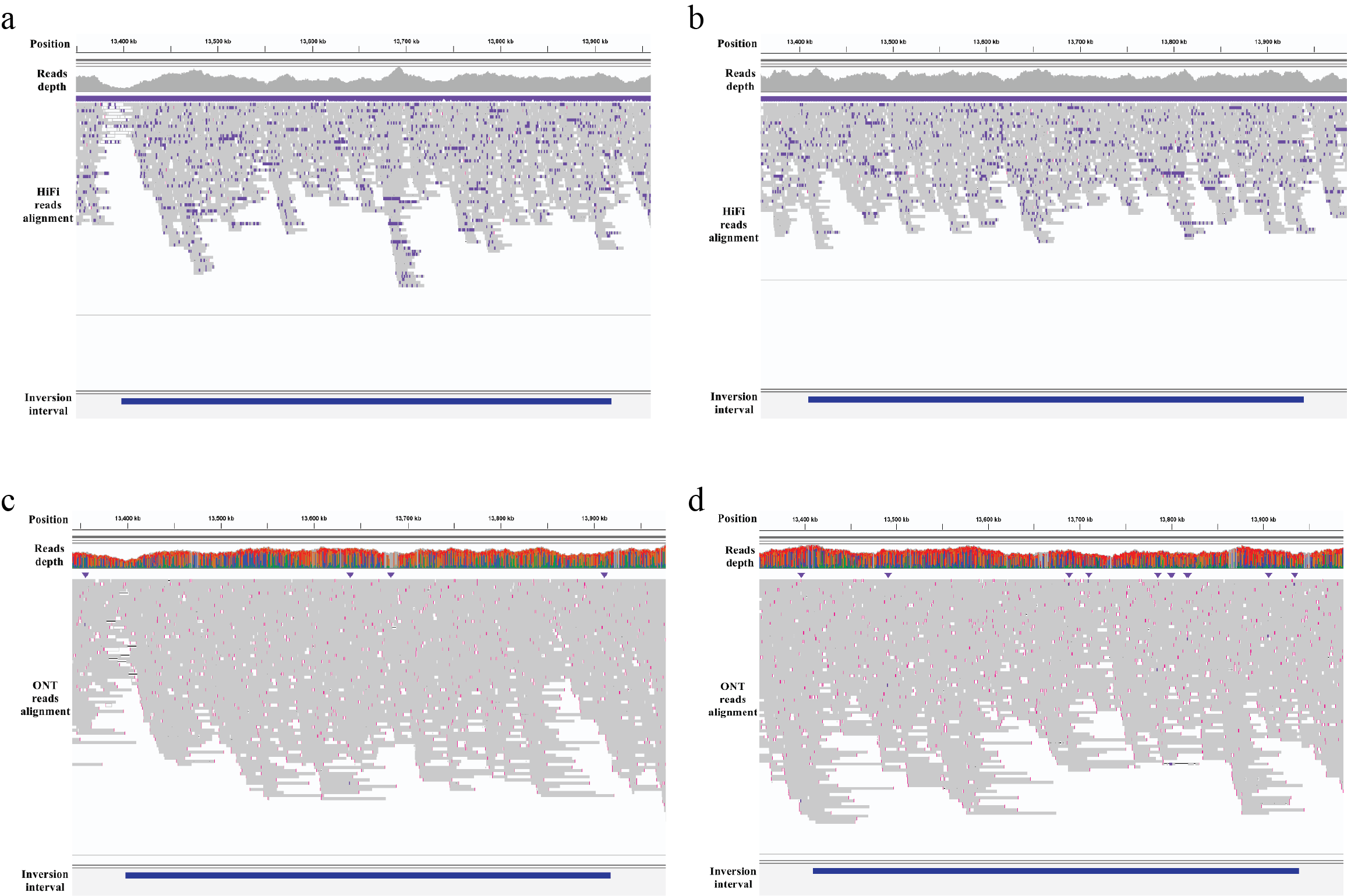


Figure S8. Read alignment results visualized in IGV. Each subfigure includes four tracks from top to bottom: genomic position, read depth, read alignments, and the inversion interval (indicated by a blue ribbon). a. HiFi reads aligned to the HapA genome; b. HiFi reads aligned to the HapB genome; c. ONT reads aligned to the HapA genome; d. ONT reads aligned to the HapB genome.


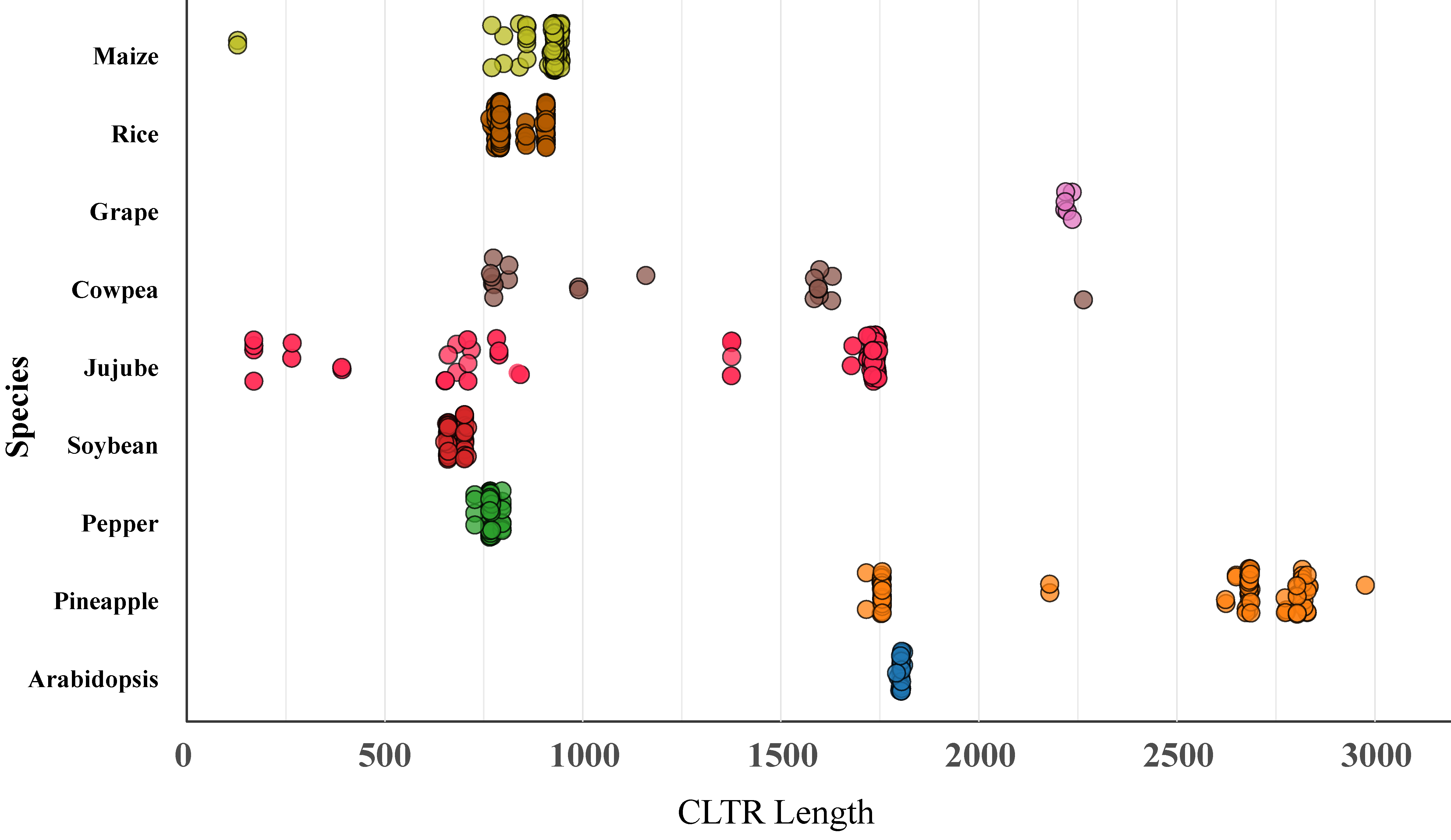


Figure S9. Length distribution of CLTRs across nine plant species.


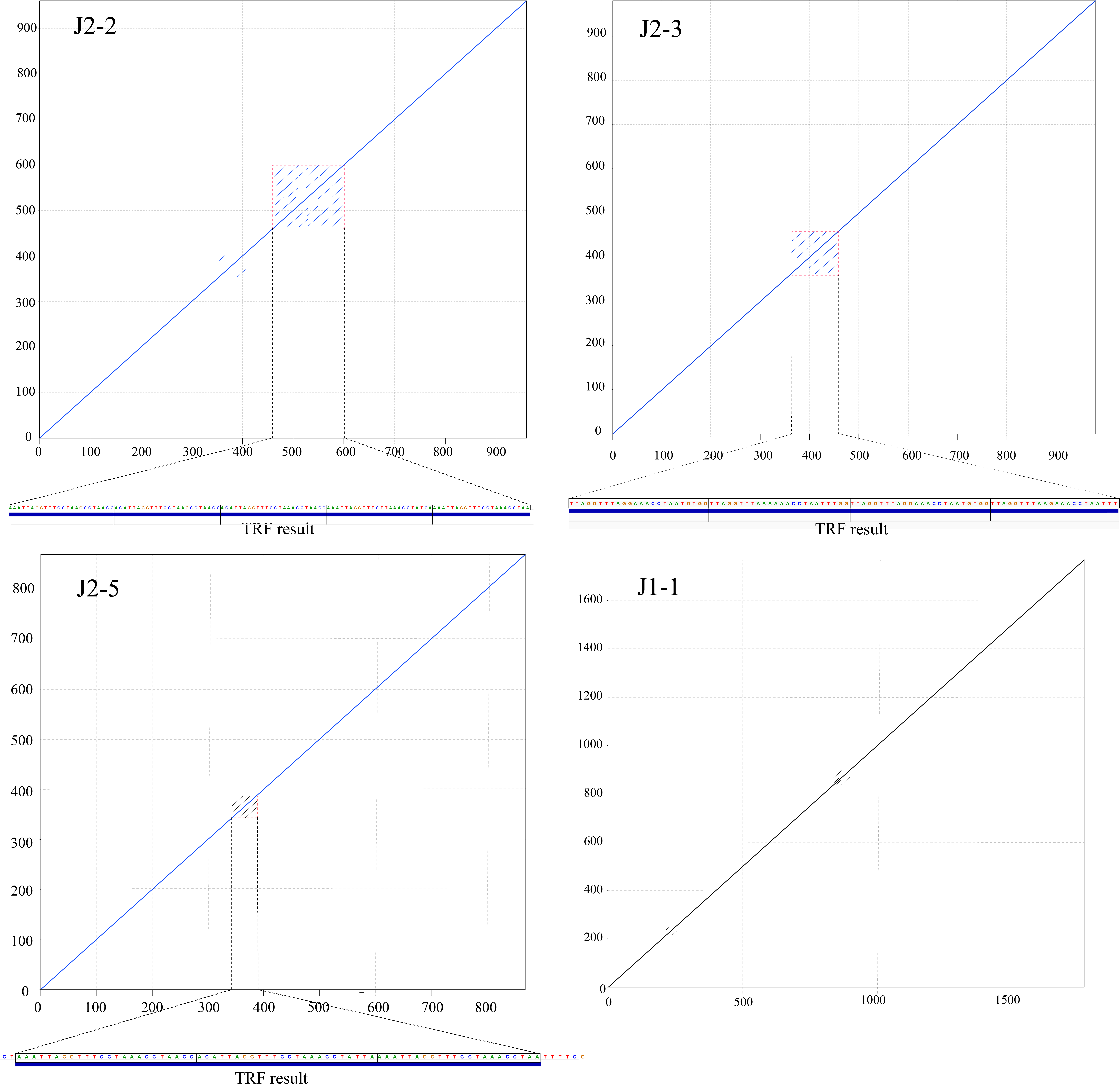


Figure S10. Self-alignment and TR identification of four CLTRs: J2-2, J2-3, J2-5, and J1-1. The collinearity plots display the self-alignment of each CLTR, while the sequence panels below show TRs identified by trf-finder. Individual TR units are delineated by boxed lines.
